# LINC01089 is a tumor-suppressive lncRNA in gastric cancer and it regulates miR-27a-3p/TET1 axis

**DOI:** 10.1186/s12935-020-01561-9

**Published:** 2020-10-16

**Authors:** Xufeng Guo, Ming Li

**Affiliations:** 1 Cancer Center, Renmin Hospital of Wuhan University, Wuhan, 430000 Hubei China; 2 Department of Gastroenterology, Renmin Hospital of Wuhan University, Zhangzhidong Road, Wuchang District, Wuhan, 430000 Hubei China

**Keywords:** LINC01089, miR-27a-3p, TET1, Gastric cancer

## Abstract

**Background:**

Gastric cancer (GC) is one of the most common malignancies around the world. Recently, the role of long non-coding RNA (lncRNA) in cancer biology has become a hot research topic. This work aimed to explore the biological function and underlying mechanism of LINC01089 in GC.

**Methods:**

Quantitative real-time polymerase chain reaction (qRT-PCR) was employed to investigate the expression of LINC01089 in GC tissues and cells. The relationship between the expression level of LINC01089 and the clinicopathological parameters of GC was assessed. Cell models of LINC01089 overexpression, LINC01089 knockdown, miR-27a-3p overexpression, and miR-27a-3p inhibition were established by transfection. CCK-8 assay, BrdU assay, and Transwell assay were utilized to investigate the malignant biological behaviors of GC cell lines after transfection. Dual luciferase activity reporter assay, Pearson’s correlation analysis, and Western blot were utilized to the regulatory relationships among LINC01089, miR-27a-3p and tet methylcytosine dioxygenase 1 (TET1).

**Result:**

LINC01089 down-regulation was observed in GC tissues and cell lines. Low expression level of LINC01089 in GC tissues was markedly linked to larger tumor size, higher T stage, as well as lymphatic metastasis of the patients. Functional experiments implied that LINC01089 overexpression impeded the proliferation, migration, as well as invasion of GC cells, whereas LINC01089 knockdown promoted the above malignant phenotypes. Additionally, up-regulation of miR-27a-3p was also observed in GC tissues. Functional experiments also showed that, miR-27a-3p overexpression boosted the malignant biological behaviors of GC cells; on the contrast, these phenotypes were impeded by miR-27a-3p inhibition. Moreover, LINC01089 interacted with and repressed miR-27a-3p, and miR-27a-3p antagonized the impact of LINC01089 on GC cells. Additionally, TET1 was verified as a target gene of miR-27a-3p, and could be positively regulated by LINC01089.

**Conclusion:**

LINC01089 impedes the proliferation, migration, and invasion of GC cells by adsorbing miR-27a-3p and up-regulating the expression of TET1.

## Background

Gastric cancer (GC), a common gastrointestinal malignancy, is one of the most deadly cancer around the world [1]. Except in Japan and South Korea, in most countries and regions in the world, most GC patients are with advanced stage cancer when diagnosed, which indicates adverse prognosis [2]. Unfortunately, GC is considered as a cancer resistant to radiotherapy and chemotherapy [3, 4]. Consequently, it is essential to understand the molecular mechanisms of GC progression, and to find novel biomarkers and therapeutic targets.

Long non-coding RNAs (lncRNAs) do not have the ability of coding protein due to the lack of open reading frame, and previously they were regarded as transcriptional “noise” or “garbage” [5]. However, accumulating studies have proved that lncRNAs have a prominent impact on transcriptional and post-transcriptional regulation on gene expression, including regulating the recruitment of transcription factors, chromatin remodeling, histone modification, pre-mRNA splicing and so on [6]. Moreover, it has been reported that many lncRNAs are crucial regulators in the tumorigenesis and cancer progression [7–9].

Some recent studies have shown that LINC01089 has tumor-suppressive effects in some cancers, like breast cancer and gliomas [10–12]. But the role of LINC01089 in GC progression and its regulatory mechanism remain unclear. In this study, it is confirmed that down-regulation of LINC01089 can also be found in GC. It is also demonstrated that LINC01089 suppresses the malignant biological behaviors of GC cells by targeting miR-27a-3p and up-regulating tet methylcytosine dioxygenase 1 (TET1). This study provides new clues for the diagnosis and therapy for GC.

## Materials and methods

### Clinical samples and ethics statement

87 cases of GC tissues/adjacent normal tissues were obtained during surgery and stored at − 196 °C in liquid nitrogen within 10 min. The matched normal tissues were collected at a distance of > 5 cm from the tumor tissues, and all tissues were identified histologically. The patients enrolled were diagnosed by gastroscope and biopsies. 55 patients were male and 32 patients were female. The median age of the patients at diagnosis was 55.4 years old (range 32–71 years old). The median diameter of the tumor was 4.3 cm (range 1.2–7.2 cm). The patients didn’t receive neoadjuvant chemotherapy or radiotherapy before the surgery. The procedures of human tissue collection and use were approved by the Research Review Board of Remin Hospital of Wuhan University. All the patients involved signed informed consent.

### Cell lines and cell culture

Human GC cell lines AGS, BGC-823, HGC-27, MGC-803, SGC-7901 cells, and immortalized gastric epithelial cell line GES-1 cells were bought from Type Culture Collection of the Chinese Academy of Sciences (Shanghai, China). Cells were cultivated in Dulbecco’s modified Eagle’s medium (DMEM, Invitrogen, Carlsbad, CA, USA) containing 10% fetal bovine serum (FBS, Invitrogen, Carlsbad, CA, USA), 100 U/ml penicillin, and 100 μg/ml streptomycin (Hyclone, Logan, UT, USA) in an incubator at 37 °C with 5% CO_2_. The medium was changed every 3–4 days. Subculture was performed with 0.25% trypsin (Hyclone, Logan, UT, USA).

### Cell transfection

MGC-803 and AGS cells were sub-cultured into a 60 mm culture plate, cultured for 24 h, and then transfected. LINC01089 overexpression plasmid, LINC01089 siRNA, miR-27a-3p mimics, miR-27a-3p inhibitors, and their negative controls were bought from GenePharma Co., Ltd. (Shanghai, China). All the procedures followed the instructions of Lipofectamine™ 2000 (Invitrogen; Thermo Fisher Scientific, Inc., Carlsbad, CA, USA).

### Quantitative real-time polymerase chain reaction (qRT-PCR)

TRIzol Regent (Invitrogen, Carlsbad, CA, USA) was employed to extract total RNA from tissues and cells. SuperScript First Strand cDNA System (Invitrogen, Carlsbad, CA, USA) was utilized to reverse transcribe 1 μg of total RNA into complementary DNA (cDNA). With cDNA as template, qRT-PCR was performed on ABI 7500 Fast Real-Time PCR System (Applied Biosystems, Waltham, MA, UK) with SYBR^®^PremixExTaqTM kit (Takara, Dalian, China). The relative expressions of LINC01089, miR-27a-3p, and TET1 were measured employing 2^−ΔΔCT^ method, with GAPDH or U6 as endogenous controls. The primers were shown in Table 1.

**Table 1:** Primer sequence

Gene	Sequence
LINC01089	F: 5′-GCAGTAAACAGTCCTCAGCGAAG-3′
	R: 5′-CGGTGCCATGGAGTCTAGAAGAT-3′
miR-27a-3p	F: 5′-TTCACAGTGGCTAAGTTCCGC-3′
U6	F: 5′-ATTGGAACGATACAGAGAAGATT-3′
	R: 5′-GGAACGCTTCACGAATTTG-3′
TET1	F: 5′-CGCTACGAAGCACCTCTCTTA-3′
	R: 5′-CTTGCA TTGGAACCGAATCATTT-3′
GAPDH	F: 5′-GAGTCAACGGA TTGGTCGT-3′
	R: 5′-TTGATTTGGAGGATCTCG-3′

*F* forward, *R* reverse, *GAPDH* glyceraldehyde phosphate dehydrogenase

### Cell counting kit-8 (CCK-8) experiment

Cells in the exponential growth phase were prepared into single cell suspension, and the density was regulated after the cells were counted. 1000 cells resupended in 100 μL medium per well were added into each well of a 96-well plate, and 6 duplicate wells were set in each group. 1 d later, 10 μL CCK-8 solution (Dojindo, Kumamoto, Japan) was added into each well. Moreover, a blank control well that only contained the medium and CCK-8 solution was set. 2 h after incubation, a microplate reader was utilized to determine the absorbance (A) value of every well at a wavelength of 450 nm. Each well plate was measured every 24 h for successive 4 d. The cell growth curve was plotted with time as the abscissa and A_450nm_ values as the ordinate.

### BrdU experiment

MGC-803 and AGS cells were sub-cultured in a 24-well plate. After the confluence of cells arrived at about 80%, 10 μM BrdU solution (RiboBio, Guangzhou, China) was added and the cells were incubated at 37 °C for 4 h. Subsequently, the medium was discarded. After the cells were washed 3 times with PBS, 4% paraformaldehyde was added to fix the cells for 10 min. After the cells were rinsed for 3 times with PBS, HCl solution was added to denature the DNA. Subsequently, 1% bovine serum albumin (Beyotime, Shanghai, China) was added, and the unspecific antigens were blocked for 1 h. Then BrdU monoclonal antibody (ab6326, 1: 300, Abcam, Cambridge, UK) was added into each well, and the cells were incubated at 4 °C overnight. After that, secondary antibody (Beyotime, Shanghai, China) was added to incubate the cells in dark for 2 h at room temperature. Subsequently, cell nuclei were stained with DAPI solution (RiboBio, Guangzhou, China), and then the cells were observed using a fluorescent microscope. 10 non-overlapping fields were randomly chosen. The number of BrdU positive cells was then counted and the average value was taken.

### Wound healing experiment

Transfected MGC-803 and AGS cells were inoculated in 12-well plates, respectively. When the cells grew to about 80% confluence, a cell-free area was made by scratching vertically with the tip of a 200 μl pipette. Then PBS was used to gently wash the wells and the wound was observed under a microscope and photographed. Then serum-free medium was added into the wells, the cell culture was continued. 24 h later, the wound was observed again. Then the wound healing of the cells was compared.

### Tanswell experiment

Migration experiments were performed employing Transwell system (EMD Millipore Corporation, Billerica, MA, USA). After the GC cells were dispersed with 0.25% trypsin, the GC cells were centrifuged, re-suspended with serum-free medium, and inoculated into the upper chamber in 24-well plates, which contained complete medium (600 μ/l per well). The cells were cultured in 37 °C for 24 h. After that, the cells on the upper surface of membrane were gently wiped off with a cotton swab, and the cells adherent to the lower surface of the membrane were then fixed with 95% ethanol, and subsequently stained with 0.1% crystal violet for 10 min. Under the microscope, cells in 5 fields of view were randomly chosen, photographed, and counted. In the invasion experiment, a layer of Matrigel (BD Biosciences, CA, USA) was employed to cover the bottom of the Transwell chamber. Moreover, the other procedures were the same as the migration experiment.

### Western blot

Cells were immersed in pre-cooled RIPA lysis buffer (Solarbio, Beijing, China), and placed on ice for 30 min. After centrifugation, the supernatant was collected. After quantifying the protein using BCA reagent (Pierce, Rockford, IL, USA), the samples were added with loading buffer, and denatured in boiling water. After the separation of protein samples by SDS-PAGE, the proteins were transferred to the polyvinylidene fluoride (PVDF) membrane (Millipore, Bedford, MA, USA). Next, the membrane was incubated in blocking buffer (5% skimmed milk) for 1 h at room temperature, and subsequently washed with TBST solution. Next, the membrane was incubated overnight with primary antibodies at 4 °C. The primary antibodies included anti-TET1 antibody (Abcam, ab220867, 1: 2000, Cambridge, UK) and anti-GAPDH antibody (endogenous control, Abcam, ab181602, 1: 3000, Cambridge, UK). After that, the PVDF membrane was rinsed with TBST solution, then incubated with secondary antibodies (Abcam, ab125900, 1: 2000, Cambridge, UK) for 1 h at room temperature. After the PVDF membrane was rinsed with TBST solution again, high-sensitivity ECL kit (Beyotime, Shanghai, China) was utilized to chemiluminescence development.

### Dual luciferase reporter gene

Luciferase reporter assay was carried out by with dual-luciferase reporter assay system (Promega, Madison, WI, USA). The target fragments of wild type LINC01089/3′UTR of TET1 and mutant LINC01089/3′UTR of TET1 were synthesized and integrated into psi-CHECK2 reporter vector (Promega, Madison, WI, USA) to construct the recombinant reporter vectors. The recombinant reporter vectors were co-transfected into HEK293T cells with miR-27b-3p mimics or control microRNAs. 48 h after transfection, luciferase activity was determined according to the manufacturer’s instructions.

### RNA Immunoprecipitation (RIP) assay

EZ-Magna RNA binding protein immunoprecipitation kit (Millipore, Billerica, MA, USA) was employed to perform RIP experiment, and the cells were harvested and resuspended in RIP lysis buffer. The cell extractives were subsequently incubated with RIP buffer which contains magnetic beads conjugated anti-Ago2 antibody or mouse IgG overnight. After the magnetic beads were washed 3 times, they were incubated with proteinase K. Subsequently, total RNA was extracted using TRIzol reagent (Invitrogen, Carlsbad, CA, USA). Finally, the relative expression of LINC01089 was determined by qRT-PCR analysis.

### Statistical analysis

Statistical analysis was carried out by SPSS 20.0 (IBM Corp., Armonk, NY). Each experiment was repeated independently at least 3 times. The results were shown as mean ± standard deviation (SD). One-Sample Kolmogorov–Smirnov test was performed to analyze whether the data are normally distributed or skewed distributed. For normally distributed data, comparisons between two groups were performed using student’s t test. The comparisons among three or more groups were performed with One way analysis of variance. If the data showed significant difference, Tukey’s post hoc test analysis was perfomred to examine the difference between two groups. For skewed distributed data, the comparison between two groups was performed by paired sample Wilcoxon signed-rank test. Chi square test was utilized to evaluate the relationships between the expression levels of LINC01089 and clinicopathological parameters of GC patients. Pearson’s correlation analysis to analyze the correlations among LINC01089, miR-27a-3p and TET1 in GC tissues. *P* < 0.05 was thought to obtain statistical significance.

## Result

### The expression of LINC01089 in GC and its clinical significance

**Fig. 1:**
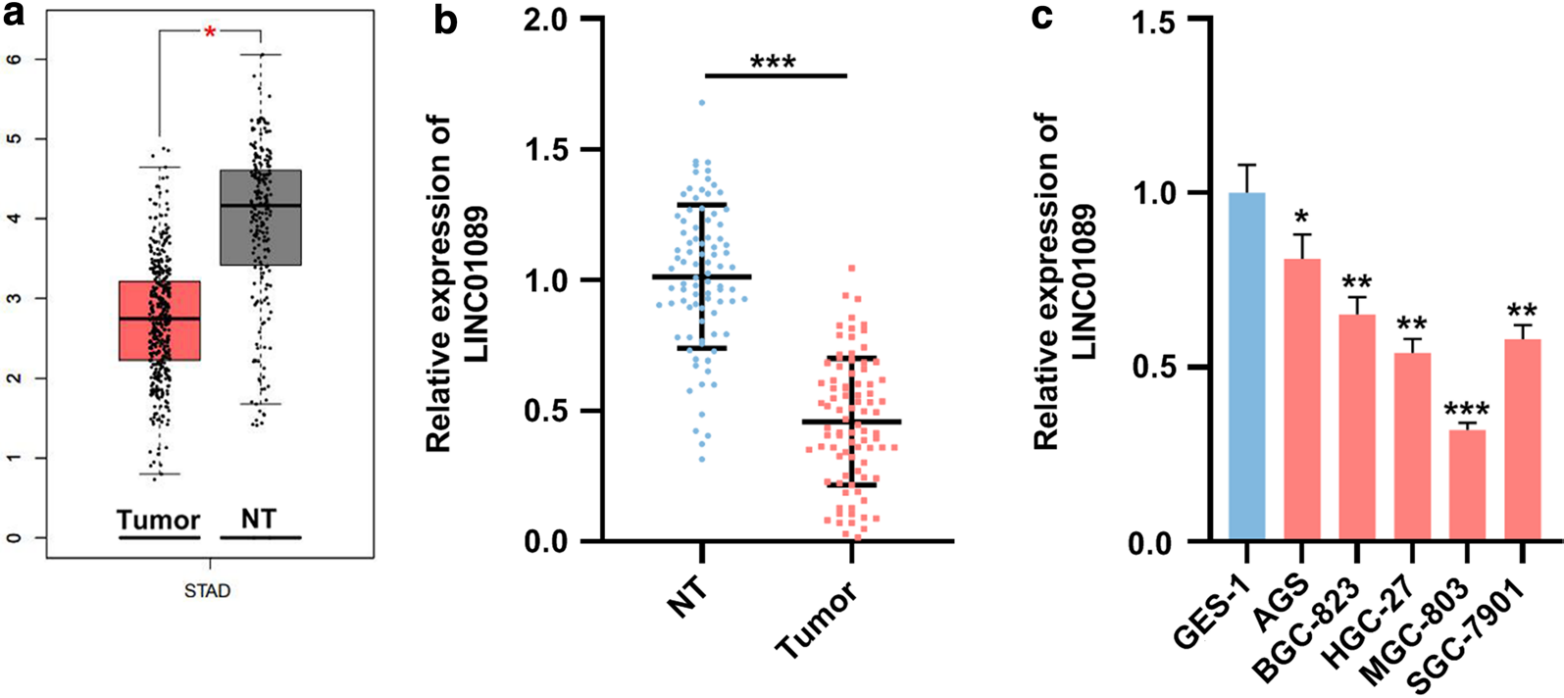
LINC01089 was up-regulated in GC samples and GC cell lines. **a** Bioinformatics analysis (GEPIA database) of the expression of LINC01089 in GC tissues and normal gastric tissues. (NT, non-tumor tissues). **b** qRT-PCR was utilized to investigate the expression level of LINC01089 in paired tumor tissue and non-tumor tissue adjacent to the tumor (n = 87). (NT, non-tumor tissues). **c** qRT-PCR was utilized to investigate the expression levels of LINC01089 in human normal gastric epithelial cell (GES-1 cells) and five GC cells (AGS, BGC-823, HGC-27, MGC-803, SGC-7901 cells). * symbolizes *P* < 0.05, ** symbolizes *P* < 0.01, and *** symbolizes *P* < 0.001

In order to study the expression characteristics of LINC01089 in GC tissues, the GEPIA database (http://gepia.cancer-pku.cn/) was used to perform bioinformatics analysis, and it was found that LINC01089 was differentially expressed in GC tissues and normal tissues; moreover, its expression was markedly lower in GC tissues (Fig. 1a). qRT-PCR was then employed to detect the expression of LINC01089 in paired tumor tissues and adjacent non-tumor tissues from GC patients. As shown, compared with adjacent tissues, the expression of LINC01089 in GC tissues was markedly down-regulated (Fig. 1b). Moreover, qRT-PCR was employed to investigate the expression of LINC01089 in immortalized gastric epithelial cells (GES-1 cells) and GC cells (AGS, BGC-823, HGC-27, MGC-803, SGC-7901 cells). These results illustrated that compared with GES-1 cells, the expression of LINC01089 in the above five GC cells were markedly down-regulated (Fig. 1c). Moreover, it was demonstrated that low expression of LINC01089 was markedly linked to tumor size, T stage, as well as lymphatic metastasis (Table 2). The results illustrated that LINC01089 has a tumor suppressive impact on GC.

**Table 2:** Correlations between LINC01089 expression and clinical characteristics in GC patients

Pathological indicators	Number of patients	Relative expression of LINC01089		*P* value
		High expression	Low expression	
All cases	87	41	46	
Age (years)				
< 57	48	22	26	0.789
≥ 57	39	19	20	
Gender				
Female	32	13	19	0.354
Male	55	28	27	
Tumor size (cm)				
< 5	46	27	19	0.022*
≥ 5	41	14	27	
T stage				
1–2	48	28	20	0.020*
3–4	39	13	26	
Lymphatic metastasis				
Positive	20	5	15	0.024*
Negative	67	36	31	
Histologic differentiation				
Well/moderately	57	26	31	0.667
Poor	30	15	15	

* *P *< 0.05

### LINC01089 impedes the proliferation, migration, and invasion of GC cells

**Fig. 2:**
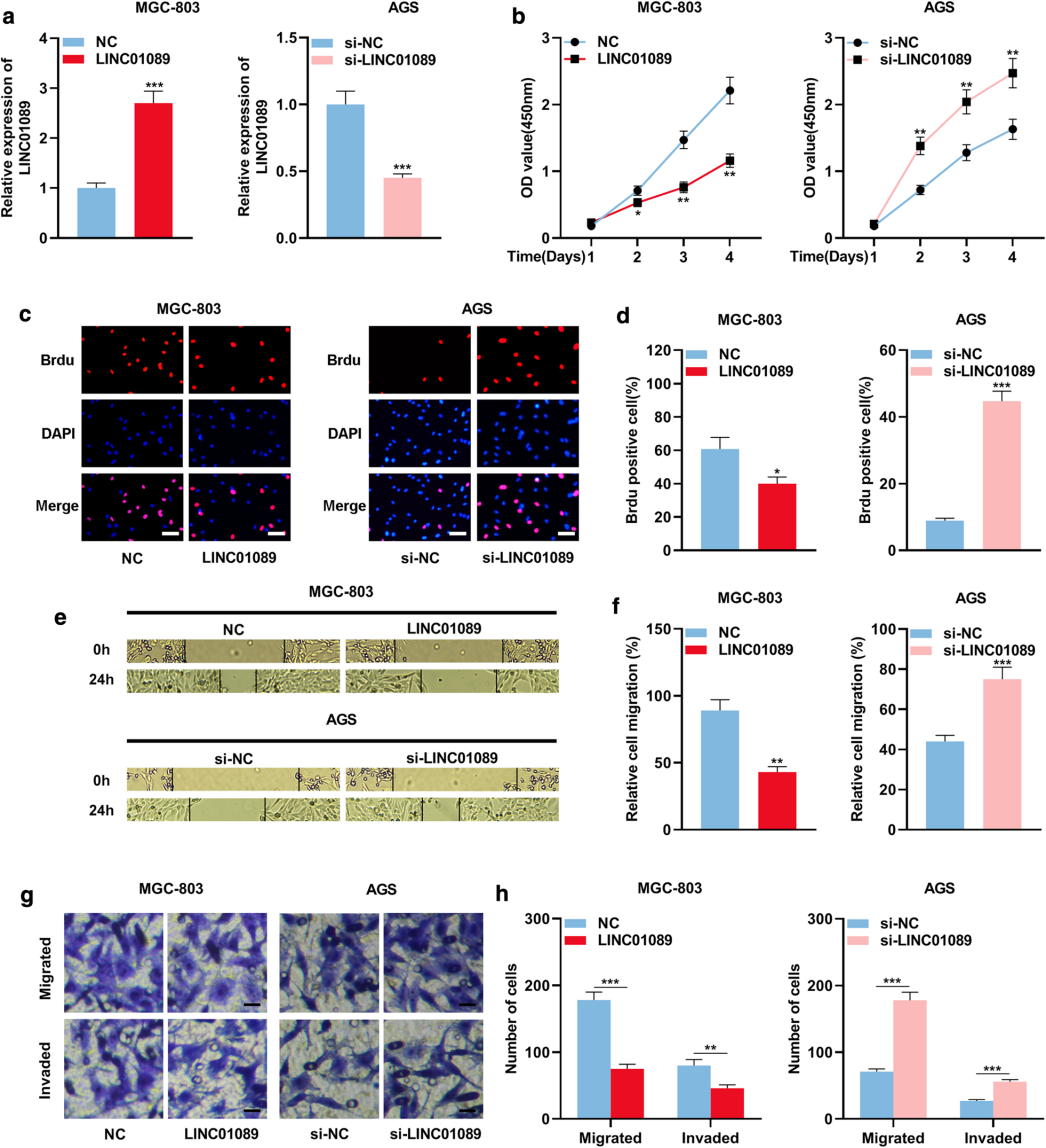
LINC01089 impeded the proliferation, migration, and invasion of GC cells. **a** qRT-PCR was utilized to investigate the expression of LINC01089 in MGC-803 cells and AGS cells after they were transfected with LINC01089 overexpression plasmid and siRNA targeting LINC01089. **b** CCK-8 method was utilized to investigate the proliferation of GC cells after transfection. **c**, **d** BrdU assay was utilized to investigate the proliferation of GC cells after transfection (scale bar = 20 µm). **e**, **f** Wound healing assay was utilized to investigate the migration of GC cells after transfection. **g**, **h** Transwell assay was utilized to investigate the migration and invasion of GC cells after transfection (scale bar = 20 µm). * symbolizes *P* < 0.05, ** symbolizes *P* < 0.01, and *** symbolizes *P* < 0.001

The expression level of LINC01089 was the lowest in MGC-803 cells in five types of cells, whereas its expression level was the highest in AGS cells. Consequently, the LINC01089 overexpression plasmid was transfected into MGC-803 cells; the expression of LINC01089 in AGS cells was knocked down with siRNA. Additionally, qRT-PCR verified that the overexpression and knockdown models were successfully constructed (Fig. 2a). Then it was confirmed through CCK-8 experiment and BrdU experiment that compared with the control group, LINC01089 overexpression impeded the proliferation of MGC-803 cells (Fig. 2b–d). To explore the effects of LINC01089 on the GC cell migration and invasion, we firstly performed wound healing experiment, and the results suggested that overexpression of LINC01089 led to a significant decrease in the migration ability of MGC-803 cells (Figs. 2e, f). In addition, Transwell experiments were performed. The results illustrated that LINC01089 overexpression markedly impeded the number of migration and invasion of MGC-803 cells (Figs. 2g, h). In contrast, knockdown of LINC01089 in AGS cells led to the opposite effects (Fig. 2b–h).

### Targeting relationship between LINC01089 and miR-27a-3p in GC cells

**Fig. 3:**
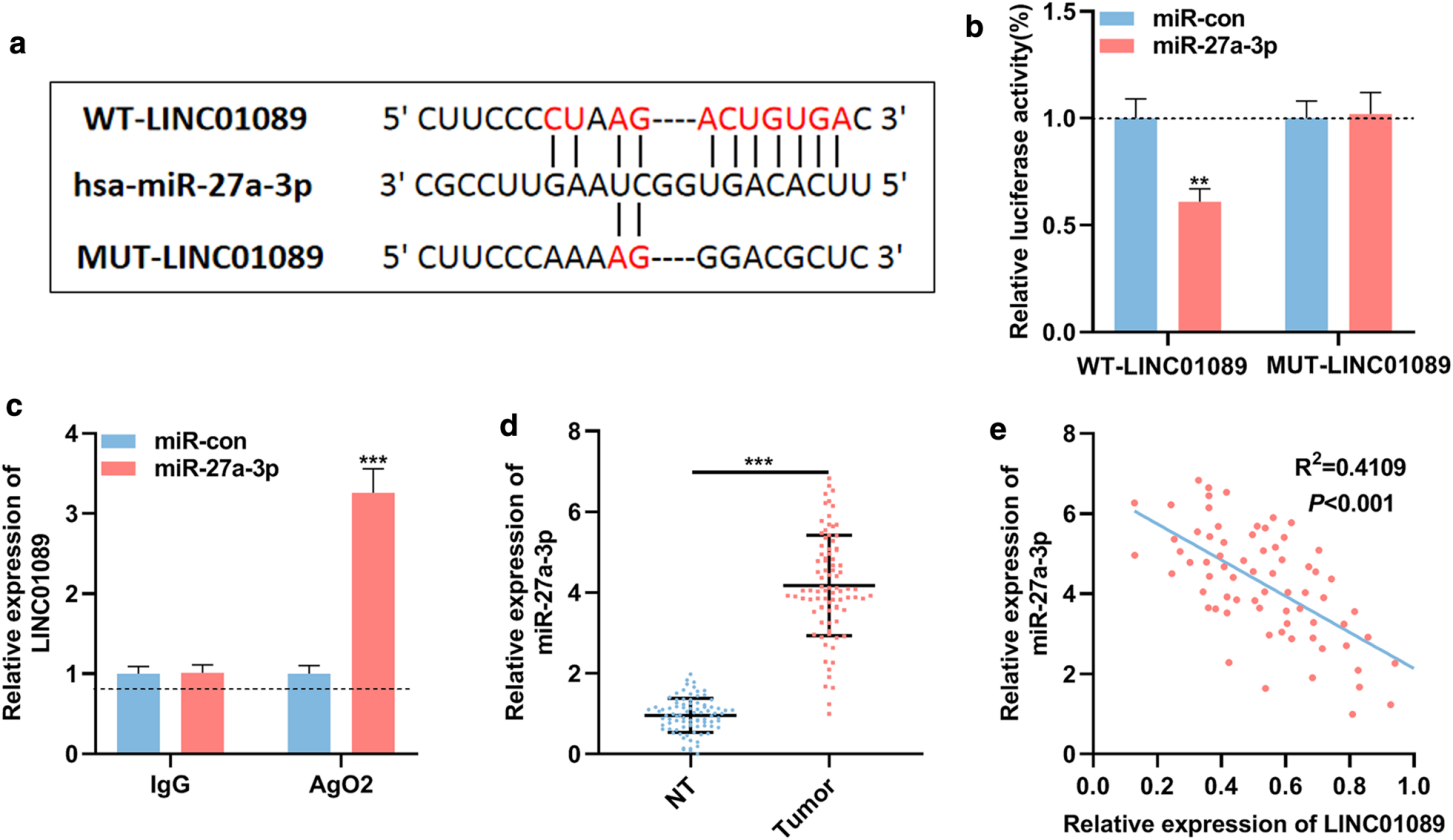
miR-27a-3p was the target of LINC01089 in GC. **a** WT-LINC01089 and MUT-LINC01089 luciferase reporter vectors containing the binding site for miR-27a-3p were constructed. **b** miR-27a-3p mimics or control miRNA were co-transfected with WT-LINC01089 or MUT-LINC01089 into 293T cells, and then the luciferase activity of each group was measured. **c** RIP assay confirmed that LINC01089 and miR-27a-3p were directly interacted in the RISC complex. **d** qRT-PCR was utilized to investigate the expression level of miR-27a-3p in paired tumor tissues and adjacent non-tumor tissues. (NT, non-tumor tissues). **e** Pearson’s correlation analysis indicated that LINC01089 and miR-27a-3p expression levels were negatively correlated in GC tissues. ** symbolizes *P* < 0.01, and *** symbolizes *P* < 0.001

Next, bioinformatics analysis was performed with StarBase database and LncBase Predicted v2 to identify candidate miRNAs targeted by LINC01089, and it showed that miR-27a-3p and LINC01089 had complementary binding sites (Additional file 1: Figure S1A, Fig. 3a). In order to further assess the relationship between LINC01089 and miR-27a-3p in GC, the luciferase reporter vector containing the predicted binding site and its mutant were constructed (Fig. 3a). Dual-luciferase reporter assay indicated that, co-transfection of miR-27a-3p mimics markedly reduced the luciferase activity of the WT-LINC01089 vector, but had no significant impact on the luciferase activity of the MUT-LINC01089 vector (Fig. 3b). Subsequently, RIP experiments confirmed that compared with IgG group, LINC01089 and miR-27a-3p were enriched in Ago2-containing microribonucleoproteins (Fig. 3c). Moreover, a significant negative correlation could be found between the expressions of LINC01089 and miR-27a-3p in GC tissues (Fig. 3d). As shown (Fig. 3e), the expression level of miR-27a-3p in GC tissues was markedly higher than that in non-tumor tissues adjacent to the cancer. These results showed that the dysregulation of LINC01089 contributed to the up-regulation of miR-27a-3p in GC tissues. Additionally, high miR-27a-3p expression in GC tissues was significantly associated with higher T stage, suggesting it might promoted the progression of GC (Additional file 2: Table S1).

### MiR-27a-3p facilitates the proliferation, migration, and invasion of GC cells

**Fig. 4:**
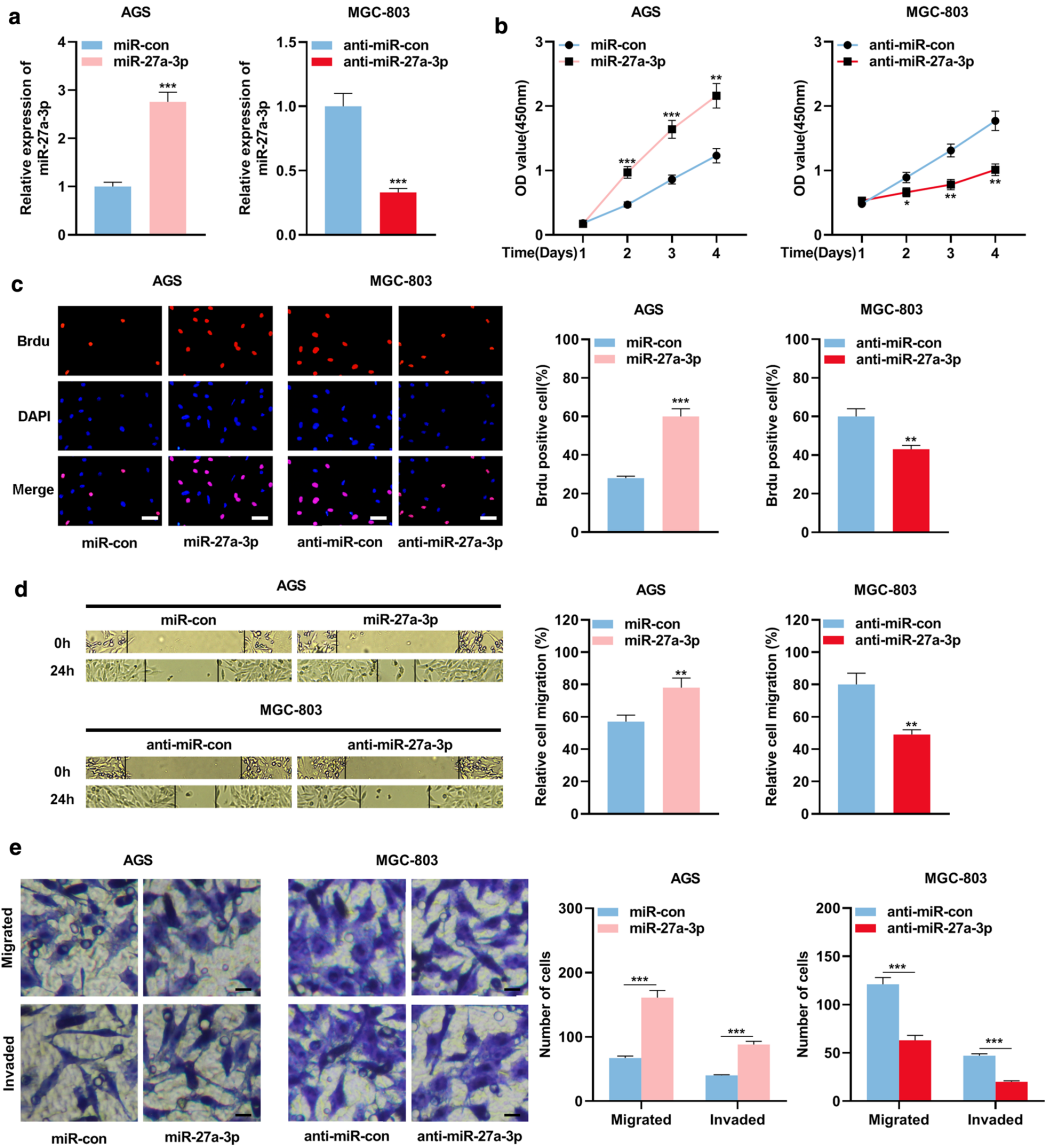
miR-27a-3p played a tumor suppressive role in GC. **a** qRT-PCR was utilized to investigate the relative expression of miR-27a-3p in AGS cells transfected with miR-27a-3p mimics and MGC-803 cells with anti-miR-27a-3p. **b**, **c** CCK-8 assay and BrdU experiment were utilized to detect the proliferation of GC cells after transfection (scale bar = 20 µm). **d** Wound healing assay was utilized to investigate the migration of GC cells after transfection. **e** Transwell assay was utilized to investigate the migration and invasion of MGC-803 and AGS cells after transfection (scale bar = 20 µm). * symbolizes *P* < 0.05, ** symbolizes *P* < 0.01, and *** symbolizes *P* < 0.001

As mentioned above, miR-27a-3p was highly expressed in GC tissues and was negatively correlated with LINC01089. To figure out the biological role of miR-27a-3p in GC, miR-27a-3p mimics were transfected into AGS cells, and anti-miR-27a-3p was transfected into MGC-803 cells, and qRT-PCR was utilized to verify the success of transfection (Fig. 4a). Cell proliferation, migration and invasion were assessed by CCK-8 experiment, BrdU experiment, wound healing experiment and Transwell experiment, respectively. It was demonstrated that up-regulating miR-27a-3p facilitated AGS cell proliferation, migration, and invasion, whereas inhibiting miR-27a-3p expression had the opposite impact on MGC-803 cells (Fig. 4b–e).

### TET1 as a target of miR-27a-3p is indirectly negatively regulated by LINC01089 in GC

**Fig. 5:**
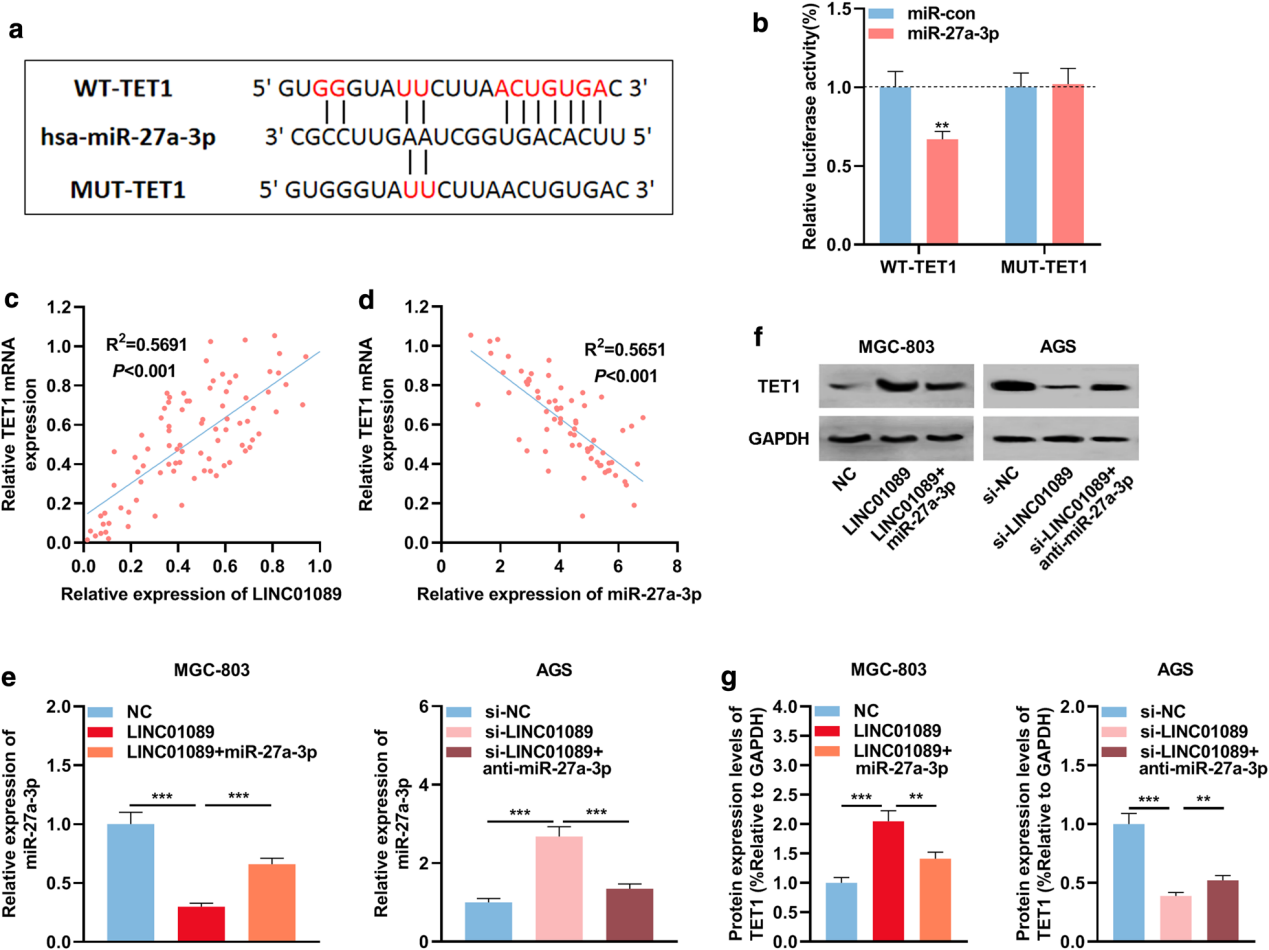
TET1 was a downstream target of miR-27a-3p in GC. **a** WT-TET1 3′UTR and MUT-TET1 3′UTR luciferase reporter vectors containing the binding site for miR-27a-3p were constructed. **b** miR-27a-3p mimics were co-transfected with WT-TET1 or MUT-TET1 into 293T cells. Then the luciferase activity of each group was measured by the double luciferase assay. **c** Pearson’s correlation analysis demonstrated that miR-27a-3p and TET1 expression levels were negatively correlated in GC tissues. **d** Pearson’s correlation analysis demonstrated that LINC01089 and TET1 expression levels were positively correlated in GC tissues. **e** miR-27a-3p mimics were transfected into MGC-803 cells with LINC01089 overexpression, and anti-miR-27a-3p was transfected into AGS cells with LINC01089 knockdown. Then qRT-PCR was utilized to detect the expression level of miR-27a-3p in GC cells. **f**, **g** Western blot was utilized to investigate the expression of TET1 protein in MGC-803 cells and AGS cells after transfection. ** symbolizes *P* < 0.01, and *** symbolizes *P* < 0.001

In order to study the mechanism of miR-27a-3p on GC cells, Starbase, TargetScan and miRTarBase were utilized to search miR-27a-3p candidate targets. It was found that TET1 was one of miR-27a-3p candidate targets (Fig. 5a). To verify whether miR-27a-3p could bind to the 3′UTR of TET1, dual luciferase reporter experiment was performed. As shown, miR-27a-3p markedly decreased the 3′UTR luciferase activity of WT TET1 reporter, whereas it did not reduce the luciferase activity of MUT TET1 reporter (Fig. 5b). Moreover, Pearson’s correlation analysis illustrated a negative correlation (R^2^ = 0.5651) between miR-27a-3p expression and TET1 expression (Fig. 5c) in GC tissues, and a positive correlation (R^2^ = 0.5691) between LINC01089 and TET1 in GC tissues (Fig. 5d). Additionally, miR-27a-3p mimics were then transfected into MGC-803 cells with LINC01089 overexpression, and anti-miR-27a-3p was transfected into AGS cells with LINC01089 knockdown to construct cells model. qRT-PCR results illustrated successful transfection (Fig. 5e). Western blot results showed that up-regulating miR-27a-3p attenuated the promotion of TET1 expression induced by LINC01089 overexpression, while the inhibitory impact of LINC01089 knockdown on TET1 expression could be reversed by anti-miR-27a-3p (Figs. 5f, g). The data suggested that TET1 was targeted by miR-27a-3p and positively regulated by LINC01089 in GC. Notably, the underexpression of TET1 in GC tissues was markedly associated with larger tumor size, lymphatic metastasis and poor differentiation of cancer tissues (Additional file 3: Table S2). These data suggested that LINC01089/miR-27a-3p could probably regulate GC progression via TET1.

### LINC01089/miR-27a-3p/TET1 axis is involved in regulating the malignant biological behaviors of GC cells

**Fig. 6:**
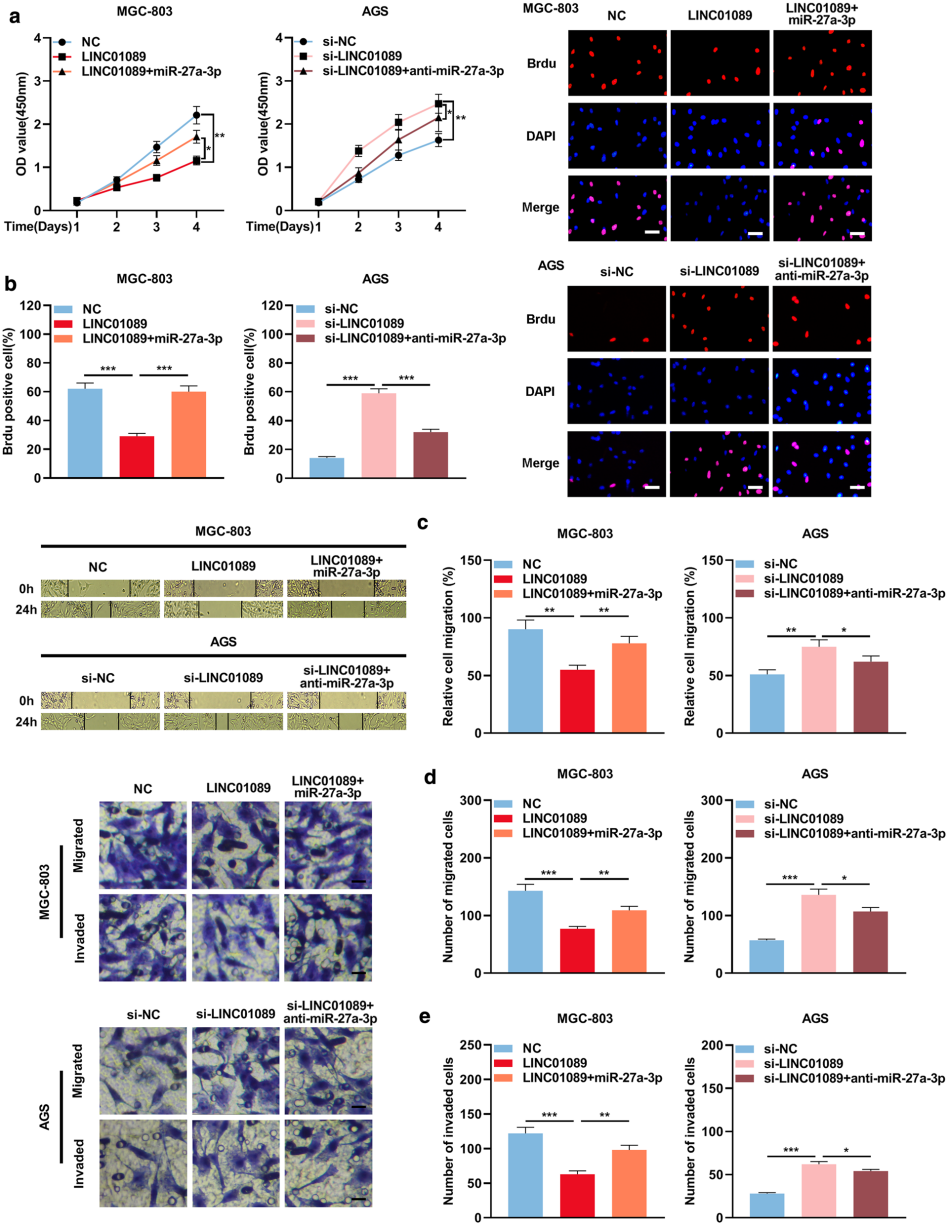
miR-27a-3p partially reversed the tumor-suppressive funciont of LINC01089 on GC cells. **a**, **b**. The proliferation of MGC-803 cells and AGS cells was investigated by CCK-8 method and BrdU experiment (scale bar = 20 µm). **c** Wound healing assay was utilized to investigate the migration of MGC-803 cells and AGS cells. **d**, **e** Transwell assay was utilized to investigate the migration and invasion of MGC-803 cells and AGS cells (scale bar = 20 µm).* symbolizes *P* < 0.05, ** symbolizes *P* < 0.01, and *** symbolizes *P* < 0.001

Subsequently, the biological behaviors of GC cells were further studied using CKK-8 experiment, BrdU experiment, wound healing experiment and Transwell experiment. The results illustrated that up-regulating miR-27a-3p reversed the inhibitory impact of over-expressing LINC01089 on proliferation (Figs. 6a, b), migration (Fig. 6c, d), as well as invasion (Fig. 6e) of MGC-803 cells, whereas the promotion effect of LINC01089 knockdown on the malignant biological behaviors of AGS cells could be attenuated by miR-27a-3p mimics (Fig. 6a–e). These results suggested that LINC01089 inhibited the proliferation, migration, and invasion of GC cells by down-regulating miR-27a-3p and up-regulating TET1 expression.

## Discussion

GC is a highly lethal malignant tumor and has caused serious public health concerns in East Asia, Eastern Europe, Central and South America [13]. Although the incidence of GC is declining, it is still the fourth most common malignant tumor around the world [14]. The molecular mechanism of gastric carcinogenesis is very complicated, and it hasn’t been elucidated [15, 16].

LncRNAs are crucial regulators participating in the tumorigenesis and cancer progression, and their dysregulation contributes to gastric carcinogenesis [17]. Accumulating lncRNAs are proven to regulate the proliferation, migration, invasion and apoptosis of GC cells. For instance, lncRNA GICHCG is over-expressed in GC and promotes the malignant biological behaviors of GC cells [2]; LncRNA ATB promotes GC growth through a miR-141-3p/TGFβ2 feedback loop [18]. Recent studies have shown that LINC01089 exerts an inhibitory effect on some cancers. In breast cancer, LINC01089 overexpression impedes cancer metastasis via regulating Wnt/β-catenin signaling [10]. Moreover, LINC01089 is down-regulated in glioma tissues, and its down-regulation predicts unfavorable prognosis of the patients; additionally, and its restoration impedes the malignant biological behaviors of glioma cells [11]. In the present study, the expression of LINC01089 in GC tissues and cells was examined. It was found that LINC01089 was markedly under-expressed in GC and the low expression of LINC01089 was associated with tumor size, T stage, as well as lymphatic metastasis. Additionally, LINC01089 overexpression impeded the proliferation, migration, and invasion of GC cells, whereas down-regulating LINC01089 promoted the above biological behaviors of GC cells. These results verify that LINC01089 is a tumor suppressor in GC.

By inhibiting translation and degrading mRNA, miRNAs participate in tumorigenesis and cancer progression [19, 20]. Previous studies illustrated that miR-27a-3p functions as an oncomiR or tumor suppressor in non-small cell lung cancer, liver cancer, esophageal cancer, and bladder cancer cells [21–24]. MiR-27a is a carcinogenic miRNA in GC, and notably, the expression level of miR-27a-3p in GC is markedly higher than that of miR-27a-5p [25, 26]. In GC, miR-27a-3p is highly expressed and has a negative correlation with survival time of the patients [27]. Moreover, miR-27a-3p boosts the proliferation, metastasis and EMT of GC cells by targeting TFPI-2 or BTG2 [26, 27]. In this study, it was found that the expression of miR-27a-3p was notably higher in GC tissues than in normal adjacent tissues. Moreover, the results of functional experiments illustrated that miR-27a-3p overexpression facilitated the proliferation and metastasis of GC cells, whereas antagonism of miR-27a-3p impeded the above behaviors of GC cells, and these results were consistent with previous report [26, 27].

It is worth noting that lncRNA can act as endogenous competitive RNA (ceRNA) to regulate gene expression indirectly, and this mechanism plays a crucial role in cancer biology [28]. For example, lncRNA PTENP1 regulates PTEN expressions by decoying miR-106b and miR-93 in GC [29]. LINC00483 is overexpressed in GC and acts as a ceRNA to regulate cell proliferation and apoptosis through sponging miR-30a-3p to activate the MAPK signaling pathway [30]. Given that LINC01089 and miR-27a-3p exert opposite biological effects in GC, it was hypothesized that LINC01089 was a molecular sponge for miR-27a-3p. In this study, through bioinformatics analysis, dual luciferase reporter assay and RIP assay, it was verified that there was a ceRNA mechanism between LINC01089 and miR-27a-3p. Importantly, functional experiments showed that miR-27a-3p could reverse the function of LINC01089 in regulating the malignant phenotyeps of GC cells. These results suggested that the down-regulation of LINC01089 in GC contributed to the dysregulation of miR-27a-3p, and the tumor-suppressive function of LINC01089 in GC was partly dependent on miR-27a-3p.

DNA methylation is an epigenetic mechanism that is crucial for controlling gene expression. TET1, a member of the TET family, induces DNA demethylation and by converting 5-methylcytosine (5mC) to 5-hydroxymethylcytosine (5hmC), and it is often down-regulated in cancers [31–33]. In GC, TET1 has been identified as a crucial modulator in both tumorigensis and cancer progression. A recent study reports that H pylori infection leads to the down-regulation of TET1, which in turn inactivate of tumor suppressor KLF4, and this process contributes to the malignant transformation of gastric epithelial cell [34]. The expression of TET1 in GC tissues is lower than that of non-tumor tissues, and knocking down TET1 enhances the proliferation, migration, as well as invasion of GC cells by regulating PTEN or p53-EZH2 pathway [35–37]. In this study, TET1 was validated as a downstream target for miR-27a-3p in GC, and this demonstration is consistent with their relationship in osteosarcoma [38]. Additionally, it was also demonstrated that TET1 could be positively regulated by LINC01089, which was dependent on miR-27a-3p. These data suggested that TET1 participated in blocking GC progression mediated by LINC01089, and the deficit of LINC01089 contributed to the down-regulation of TET1 during GC progression.

In summary, for the first time, the expression pattern, function and mechanism of LINC01089 in GC was investigated in this work. It is demonstrated that LINC01089 is lowly expressed in GC tissues and cells. Moreover, its low expression is related to the adverse clinical characteristics of GC patients. Additionally, LINC01089 impedes the proliferation, migration, as well as invasion of GC cells through regulating miR-27a-3p/TET1 axis. The present study implies that restoration of LINC01089 could be a potential strategy to treat GC.

## Conclusion

In a word, LINC01089 up-regulates the expression of TET1 at the transcriptional level through competitive binding to miR-27a-3p, thus inhibiting the growth and metastasis of GC cells. Our findings provide new mechanism for explaining the breast cancer progression as well as potential alternative for GC prevention and treatment.

## Supplementary information

**Additional file 1: Figure S1.** Hsa-miR-9-3p, hsa-miR-27b-3p, hsa-miR-124-3p, hsa-miR-148b-3p, hsa-miR-27a-3p were predicted by two bioinformatics tools (StarBase and LncBase Predicted v2) as the potential targetS of LINC01089. Among them, miR-27a-3p has the highest score. * *P* < 0.05.

**Additional file 2: Table S1.** Correlations between miR-27a-3p expression and clinical characteristics in GC patients.

**Additional file 3: Table S2.** Correlations between TET1 expression and clinical characteristics in GC patients.

## Data Availability

The data used to support the findings of this study are available from the corresponding author upon request.

## Abbreviations

GC: Gastric cancer
TET1: tet methylcytosine dioxygenase 1
GAPDH: Glyceraldehyde phosphate dehydrogenase
