# Supplementary figures and images for: LINC01089 is a tumor-suppressive lncRNA in gastric cancer and it regulates miR-27a-3p/TET1 axis
Source: Cancer Cell Int. 2020 Oct 16;20:507. doi: 10.1186/s12935-020-01561-9 (PMC7568383; doi:10.1186/s12935-020-01561-9)

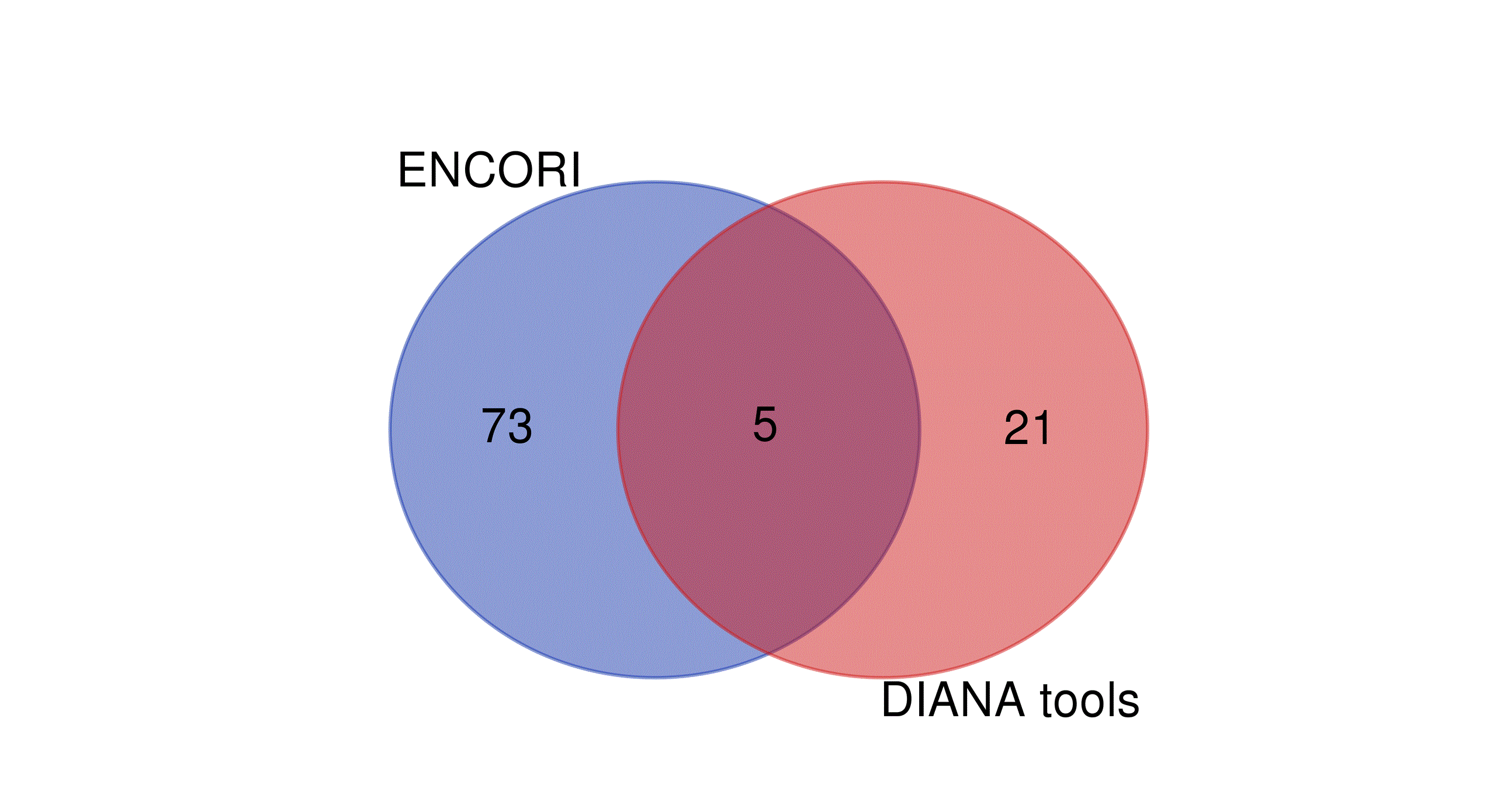

Supplement: Supplementary file 1 — Additional file 1: Figure S1. Hsa-miR-9-3p, hsa-miR-27b-3p, hsa-miR-124-3p, hsa-miR-148b-3p, hsa-miR-27a-3p were predicted by two bioinformatics tools (StarBase and LncBase Predicted v2) as the potential targetS of LINC01089. Among them, miR-27a-3p has the highest score. * P < 0.05. [file 12935_2020_1561_MOESM1_ESM.gif]
