# Supplementary material for: LINC01089 is a tumor-suppressive lncRNA in gastric cancer and it regulates miR-27a-3p/TET1 axis
Source: Cancer Cell Int. 2020 Oct 16;20:507. doi: 10.1186/s12935-020-01561-9 (PMC7568383; doi:10.1186/s12935-020-01561-9)
Supplement: Supplementary file 2 — Additional file 2: Table S1. Correlations between miR-27a-3p expression and clinical characteristics in GC patients. [file 12935_2020_1561_MOESM2_ESM.doc]

**Additional Table S1. Correlations between miR-27a-3p expression and clinical characteristics in GC patients**

| Pathological indicators | Number of patients | Relative expression of miR-27a-3p | | *P* value  (**P*<0.05) |
| --- | --- | --- | --- | --- |
|  |  | High expression | Low  expression |  |
| All cases | 87 | 50 | 37 |  |
| Age（years） |  |  |  |  |
| <57 | 48 | 25 | 23 | 0.259 |
| ≥57 | 39 | 25 | 14 |  |
| Gender |  |  |  |  |
| female | 32 | 21 | 11 | 0.241 |
| male | 55 | 29 | 26 |  |
| Tumor size (cm) |  |  |  |  |
| <5 | 46 | 23 | 23 | 0.135 |
| ≥5 | 41 | 27 | 14 |  |
| T stage |  |  |  |  |
| 1-2 | 48 | 23 | 25 | 0.046* |
| 3-4 | 39 | 27 | 12 |  |
| Lymphatic metastasis |  |  |  |  |
| positive | 20 | 9 | 11 | 0.199 |
| negative | 67 | 41 | 26 |  |
| Histologic differentiation |  |  |  |  |
| well/moderately | 57 | 30 | 27 | 0.208 |
| poor | 30 | 20 | 10 |  |

* *P*＜0.05
