# Supplementary material for: LINC01089 is a tumor-suppressive lncRNA in gastric cancer and it regulates miR-27a-3p/TET1 axis
Source: Cancer Cell Int. 2020 Oct 16;20:507. doi: 10.1186/s12935-020-01561-9 (PMC7568383; doi:10.1186/s12935-020-01561-9)
Supplement: Supplementary file 3 — Additional file 3: Table S2. Correlations between TET1 expression and clinical characteristics in GC patients. [file 12935_2020_1561_MOESM3_ESM.doc]

**Additional Table S2. Correlations between TET1 expression and clinical characteristics in GC patients**

| Pathological indicators | Number of patients | Relative expression of TET1 | | *P* value  (**P*<0.05) |
| --- | --- | --- | --- | --- |
|  |  | High expression | Low  expression |  |
| All cases | 87 | 40 | 47 |  |
| Age（years） |  |  |  |  |
| <57 | 48 | 25 | 23 | 0.205 |
| ≥57 | 39 | 15 | 24 |  |
| Gender |  |  |  |  |
| female | 32 | 14 | 18 | 0.751 |
| male | 55 | 26 | 29 |  |
| Tumor size (cm) |  |  |  |  |
| <5 | 46 | 27 | 19 | 0.012* |
| ≥5 | 41 | 13 | 28 |  |
| T stage |  |  |  |  |
| 1-2 | 48 | 23 | 25 | 0.687 |
| 3-4 | 39 | 17 | 22 |  |
| Lymphatic metastasis |  |  |  |  |
| positive | 20 | 14 | 6 | 0.014* |
| negative | 67 | 26 | 41 |  |
| Histologic differentiation |  |  |  |  |
| well/moderately | 57 | 32 | 25 | 0.009** |
| poor | 30 | 8 | 22 |  |

* *P*＜0.05，** *P*＜0.01
